# Absence of anti-HEV antibodies in donkeys in Algeria: a first serological survey

**DOI:** 10.1007/s11259-026-11094-7

**Published:** 2026-02-03

**Authors:** Soraia Rodrigues, Abdeldjalil Dahmane, Mouad Debbous, Ricardo Figueiredo, Samari Houssem, Nassiba Reghaissia, Guilherme Moreira, João R. Mesquita

**Affiliations:** 1 School of Medicine and Biomedical Sciences (ICBAS), University of Porto, 4050-313 Porto, Portugal; 2 Department of Veterinary Sciences, Faculty of Natural and Life Sciences, and Earth and Universe Sciences, Mohamed Khider University of Biskra, BP 145 RP, 0700 Biskra, Algeria; 3 Department of Veterinary Sciences, Faculty of Sciences, University of M’sila, M’sila, Algeria; 4 Institute of Veterinary and Agronomic Sciences, University of Souk Ahras, Annaba Road 41000, Souk Ahras, Algeria; 5 LAQV, REQUIMTE, Department of Chemistry and Biochemistry, Faculty of Sciences, University of Porto, Porto, Portugal

**Keywords:** Algeria, Donkey, HEV, ELISA, Hepatitis E virus

## Abstract

*Paslahepevirus balayani* (Hepatitis E virus, HEV) is an emerging zoonotic pathogen with a wide host range, yet its circulation in African equids remains poorly understood. This study provides the first serological investigation of HEV in donkeys in Algeria, aiming to assess exposure levels and identify potential infection-related risk factors. Between 2019 and 2024, 183 donkeys were sampled across three northeastern provinces (Mila, Souk-Ahras, and Tébessa), representing diverse agroecological conditions and including both working donkeys and animals used in cross-border smuggling. Individual data on age, sex, health status, body condition, movement history, and season were recorded. Sera were screened using a proprietary recombinant antigen, which is highly conserved across different HEV strains. All samples tested negative for anti-HEV antibodies. The sample size exceeded the minimum required to detect the highest seroprevalence previously reported in donkeys (12.22%), supporting the epidemiological significance of the negative findings. The absence of seropositivity may reflect limited regional exposure to known HEV reservoirs, local husbandry practices, and environmental conditions that reduce infection risk. These results suggest that HEV is currently not circulating at detectable levels in donkeys from northeastern Algeria. Broader studies, including molecular approaches and additional species, are recommended to clarify HEV ecology in the region.

## Background

*Paslahepevirus balayani* (HEV) is a small (~ 7.2 kb) non-enveloped, single-stranded, positive-sense RNA virus (Purdy et al. 2022). It is reported to cause around 20 million cases of acute hepatitis and 3450 fatalities annually worldwide (WHO 2025). HEV includes genotypes detected in humans, pigs, wild boar, deer, mongooses, rabbits, dogs, cats, horses, donkeys, camels, and other animals (Geng et al. 2011; Dong et al. 2011; Rui et al. 2020; Agabou et al. 2023; Santos-Silva et al. 2024a, b; Molini et al. 2024). Serological assays have shown evidence of HEV circulation in horses and donkeys, and while these animals are unlikely to serve as major host reservoirs, considerations toward the close contact humans have with potential sources of HEV infection are warranted, in particular for known susceptible risk groups such as immunocompromised individuals (King et al. 2018; Rui et al. 2020; Molini et al. 2024). No antibodies against HEV were detected in Namibian donkeys (Molini et al. 2024); European studies have found low but measurable seropositivity of 0.4–4.6% in horses, 3.6–11.1% in donkeys, and 3.6–8.1% mules/hinnies (García-Bocanegra et al. 2019; Caballero-Gómez et al. 2023); 13% in horses in Egypt (Saad et al. 2007)in northern China studies have found seropositivity of up to 12.22% in donkeys and 14.29% in horses(Geng et al. 2011; Rui et al. 2020);. Donkeys have a higher risk of HEV exposure compared to horses, possibly due to genetic susceptibility or environmental exposure, such as drinking from ponds and streams (Caballero-Gómez et al. 2023). Younger donkeys may be more susceptible (Rui et al. 2020). Donkeys are considered spillover hosts rather than true reservoirs for HEV, with limited evidence for their role in zoonotic transmission (Dong et al. 2011; García-Bocanegra et al. 2019; Caballero-Gómez et al. 2023).

This study aimed to assess HEV exposure in Algerian donkeys and to identify potential risk factors associated with the infection. These insights may support improved prevention strategies and help mitigate both animal health impacts and the risk of zoonotic transmission through meat consumption.

## Materials and methods

 Ethical approval for this study was granted in 2019 by the Ethics Committee of the National Veterinary School of Algiers (authorized on March, 11, 2018, Reference code 270/DPGR/2018), and some donkeys from Souk-Ahras and Tébessa were sampled in Jijel province, at the zoological park under an official permit (authorization no. 72/FDRS/2023).

A total of 183 donkeys were sampled from 2019 to 2024 from three eastern Algerian provinces: Mila, Souk-Ahras, and Tébessa, selected for their contrasting geographic and bioclimatic conditions. Mila has a Mediterranean climate, Tébessa is semi-arid with steppe landscapes, and Souk-Ahras transitions from Mediterranean to drier conditions toward the south. The study included two functional groups: donkeys used for domestic/agricultural purposes (*n* = 91) and those involved in cross-border smuggling (*n* = 92).

Sample size was calculated using the random sampling formula (Thrusfield 2018), assuming a conservative expected seroprevalence of 12.22% (Rui et al. 2020), a 5% absolute precision, and a 95% confidence level. The formula used was:

$$\:n\:=\frac{\left({Z}^{2}\:\times\:\:{P}_{exp}\:\left(1\:-\:{P}_{exp}\right)\right)}{{d}^{2}}$$

Where: *n* = sample size, *d* = desired precision (0.05), $\:{P}_{exp}$ = expected prevalence, and *Z* = 1.96 for a 95% confidence level. Our final sample of 183 animals exceeded the minimum required (165).

Blood was collected by jugular venipuncture centrifuged at 2000 g for 10 min, and sera were stored at − 20 °C until analysis. For each donkey, the following data were recorded: sex, origin, body condition (Pearson and Quassat 2000), health status, age category, sampling season, movement history, and functional use. No animals showed tick infestation at sampling. Animals were classified as clinically healthy if they exhibited calm behaviour, normal vital parameters related to rectal temperature within reference ranges, appropriate body condition, and absence of overt clinical abnormalities such as nasal discharge, abnormal respiratory effort, lameness, skin lesions, or other signs of disease upon palpation and visual inspection (Barrio et al. 2019). Movement history is related to displacement from the borderline provinces to Tunisian territory for a varied duration, or movement from the detention centres in these provinces to Zoos located in littoral provinces for at least two weeks; this information is recorded by local authorities.

To detect the specific antibodies against Hepatitis E Virus in the collected samples, the available commercial ELISA kit (MP Diagnostics™ HEV ELISA 4.0v, MP Biomedicals, Eschwege, Germany) was employed according to the instructions of the manufacturer. This ELISA utilizes a proprietary recombinant antigen, which is highly conserved across different HEV strains, to detect the presence of specific antibodies, including IgG, IgM, and IgA, against HEV. The assay has a reported sensitivity of 99.2% and specificity of 99.2% (Hu et al. 2008; Santos-Silva et al. 2024b).

The absorbance was measured as the optical density (OD). For quality control, blank values must have an absorbance of ≤ 0.100, Non-Reactive Control values must have an absorbance of ≤ 0.100, and at least 2 of the 3 Reactive Control values must have an absorbance of ≥ 0.500 and the presence or absence of antibodies specific to HEV was determined by relating the absorbance of the specimens to the cut-off value (COV) of the plate, which is calculated as:

$$COV=0.20+NRC\,\,\overline x$$

NRCx̄: Non-Reactive Control Mean.

Specimens with absorbance values less than the COV were considered non-reactive.

## Results

A total of 183 donkey serum samples were collected across various agroecological zones in northeastern Algeria (Table 1) and screened for anti-HEV antibodies using an ELISA assay. None of the samples exhibited absorbance values exceeding the cutoff threshold, and all were therefore classified as non-reactive.

**Table 1:** Description of Donkey population included in the study

Variable	Category	Number of donkeys, *n*	Distribution (%)
Donkey origin	Mila	14	7.7
	Tébessa	34	18.6
	Souk-Ahras	135	73.8
Age group	> 10 years	26	14.2
	5–10 years	62	33.9
	2–4 years	95	51.9
Sex	Male	67	36.6
	Female	116	63.4
Season	Spring	75	41
	Summer	62	33.9
	Winter	46	25.1
Movement history	No	76	41.5
	Yes	107	58.5
Body condition Scoring	Good	142	77.6
	Moderate	24	13.1
	Poor	17	9.3
Type of use	AWD	91	49.7
	SD	92	50.3
Health condition	Healthy	140	76.5
	Sick	43	23.5

*AWD* Agricultural working donkeys; *SD* Smuggling donkeys

## Discussion and conclusions

As far as the authors know, this is the first study to investigate the seroprevalence of HEV in donkeys in Algeria. Most studies report HEV seroprevalence in donkeys between 0% and 12%, with the highest rates observed in China and parts of Europe (Saad et al. 2007; Geng et al. 2011; García-Bocanegra et al. 2019; Rui et al. 2020; Caballero-Gómez et al. 2023). The absence of seropositive animals in our sample is epidemiologically meaningful, as the number of animals tested exceeded the minimum sample size required to detect the highest seroprevalence previously reported (12.22%) (Rui et al. 2020).

The commercial ELISA is highly sensitive and specific and is validated for a wide range of animal species, including donkeys, making false negatives unlikely if antibodies are present. Proper sample handling and storage protocols were followed, minimizing the risk of technical errors leading to false negatives.

The sample included both healthy (76.5%) and sick (23.5%) donkeys, as well as animals with a history of movement (58.5%). Donkeys of both sexes, various ages, and different geographical origins were also represented, minimizing sampling bias. Although very low HEV prevalence could fall below the study’s detection threshold and result in missed true positives, the sample size was sufficient to detect prevalence estimates corresponding to the highest previously reported values.

Equids (horses, donkeys) have shown very low and inconsistent seroprevalence worldwide (King et al. 2018; Caballero-Gómez et al. 2023). Thus, the absence of antibodies in Algerian donkeys is consistent with global epidemiology.

While donkeys are not considered primary HEV reservoir hosts, and existing data suggests low to no susceptibility in this species, investigating their role is still relevant in specific contexts. In regions like Namibia, donkeys may have genuinely not been exposed to HEV, resulting in no detectable antibodies (Molini et al. 2024). Regional differences in animal husbandry, water sources, and contact with other animal species can influence exposure risk (Caballero-Gómez et al. 2023; Molini et al. 2024). A significant feature of Algerian livestock systems is the predominance of small ruminants, specifically sheep and goats. While cattle and camels are also present, pig farming is rare, likely due to cultural and local practices. The absence of intensive pig farming or other known HEV reservoirs in the donkeys’ environment may have further reduced exposure in the sampled population. However, the close contact donkeys often have with humans and other animals in rural settings, their potential role as dead-end hosts or indicators of environmental contamination warrants investigation.

A limitation of this study is its exclusive focus on donkeys. Moreover, HEV is a zoonotic virus that is also found in the environment. It can be transmitted through contaminated meat or direct contact, but contamination of water (faecal-oral transmission) and crops is also a likely source of infection (WHO 2025). Therefore, the epidemiology of this virus warrants a One Health approach, both in prevention and when characterizing its presence and circulation in a population. A broader inclusion of other equids (horses, mules), co-inhabiting farm animals, or pet animals, as well as water screening, would provide greater epidemiological evidence regarding HEV circulation in the local environment, which would highlight potential differences in exposure and transmission risk to humans. Additionally, testing the same sera for other viral pathogens (e.g., vector-borne zoonotic viruses) would be relevant in a One Health perspective.

HEV is a worldwide, almost ubiquitous pathogen(King et al. 2018). In humans, a Middle East and North Africa region systematic review reports HEV IgG seroprevalence around 17–22%, which is higher than those reported in Europe (5–17%) but lower than hyperendemic Egypt, which can exceed 60% in some settings (Saade et al. 2022). In camels, there was a survey in six southern provinces of Algeria that reported a seroprevalence of 35.1% (Agabou et al. 2023). The seroprevalence in humans and camels suggests endemic circulation; however, data are still limited – in humans, age-stratified and nationwide data are limited; and in animals, besides camels in southern provinces, there were no other studies. Considering past studies, negative seroprevalence in the studied population suggests limited viral circulation, with limited exposure of donkeys to HEV, meaning this species may not play a role in the ecology or maintenance of HEV in the studied region.

## Data Availability

No datasets were generated or analysed during the current study.
